# Design of Phase III study testing buntanetap efficacy in early Alzheimer's patients based on the insights from previous studies

**DOI:** 10.1002/alz70861_108166

**Published:** 2025-12-23

**Authors:** Cheng Fang, Laurie H Sanders, Melissa Gaines, Maria L. Maccecchini

**Affiliations:** ^1^ Annovis Bio, Malvern, PA USA; ^2^ Duke University Medical Center, Durham, NC USA

## Abstract

**Background:**

By inhibiting the translation of neurotoxic aggregating proteins ‐ amyloid precursor protein, tau, alpha‐synuclein, and TDP43 etc., buntanetap restores axonal transport, lowers inflammation, and protects nerve cells from dying. Results from our phase II/III dose‐ranging study of buntanetap in mild to moderate Alzheimer’s disease (AD) patients (MMSE14‐24) showed that 30mg buntanetap improved mild AD patients (MMSE 21‐24) cognitive functions. We are validating the findings in a Phase III study in early AD patients (MMSE 21‐28).

**Method:**

A double‐blind, placebo‐controlled, phase III study is currently underway to test buntanetap’s efficacy and safety in early AD. Patients will be 1 to 1 randomized with either placebo or 30 mg QD buntanetap. A total of 760 patients will be enrolled in the US. Primary endpoints are ADAS‐Cog13 and ADCS‐iADL.

**Result:**

Biomarker data from our Phase II/III study confirmed the mechanisms of action and showed more insights into the molecular pathway, from neurotoxic proteins, synaptic function, inflammatory responses, and neurodegeneration.

The new ongoing pivotal Phase III study incorporates the pTau217 plasma biomarker into patient enrichment/validation. This study has 2 parts. Part 1 is a 6‐month study to confirm buntanetap’s symptomatic effects in improving patients’ cognition. Part 2 is an 18‐month study to test buntanetap’s potential disease modifying effects.

**Conclusion:**

Based on clinical and biomarker data from our previous studies, we designed a novel Phase 3 study to validate symptomatic improvement and potentially show disease‐modification effects of buntanetap in treating early AD patients.

